# Synergistic combination of DT‐13 and Topotecan inhibits aerobic glycolysis in human gastric carcinoma BGC‐823 cells via NM IIA/EGFR/HK II axis

**DOI:** 10.1111/jcmm.14523

**Published:** 2019-08-09

**Authors:** Xiao‐Wen Yu, Dandan Wei, Ying‐Sheng Gao, Hong‐Zhi Du, Bo‐Yang Yu, Rui‐Ming Li, Chang‐Min Qian, Xue‐Jun Luo, Sheng‐Tao Yuan, Jun‐Song Wang, Li Sun

**Affiliations:** ^1^ Jiangsu Key Laboratory for Drug Screening China Pharmaceutical University Nanjing China; ^2^ Nanjing Key Laboratory of Pediatrics Children's Hospital of Nanjing Medical University Nanjing China; ^3^ Jiangsu Collaborative Innovation Center of Chinese Medicinal Resources Industrialization, State Key Laboratory Cultivation Base for TCM Quality and Efficacy Nanjing University of Chinese Medicine Nanjing China; ^4^ Jiangsu Center for Pharmacodynamics Research and Evaluation China Pharmaceutical University Nanjing China; ^5^ School of Pharmacy Hubei University of Chinese Medicine Wuhan China; ^6^ Jiangsu Key Laboratory of TCM Evaluation and Translational Research, Department of Complex Prescription of TCM China Pharmaceutical University Nanjing China; ^7^ Tasly Research Institute Tianjin Tasly Holding Group Co. Ltd. Tianjin China; ^8^ Center for Molecular Metabolism Nanjing University of Science & Technology Nanjing China

**Keywords:** aerobic glycolysis, DT‐13, EGFR, gastric cancer, NM IIA, TPT

## Abstract

DT‐13 combined with topotecan (TPT) showed stronger antitumour effects in mice subcutaneous xenograft model compared with their individual effects in our previous research. Here, we further observed the synergistically effect in mice orthotopic xenograft model. Metabolomics analysis showed DT‐13 combined with TPT alleviated metabolic disorders induced by tumour and synergistically inhibited the activity of the aerobic glycolysis‐related enzymes in vivo and in vitro. Mechanistic studies revealed that the combination treatment promoted epidermal growth factor receptor (EGFR) degradation through non‐muscle myosin IIA (NM IIA)‐induced endocytosis of EGFR, further inhibited the activity of hexokinase II (HK II), and eventually promoted the aerobic glycolysis inhibition activity more efficiently compared with TPT or DT‐13 monotherapy. The combination therapy also inhibited the specific binding of HK II to mitochondria. When using the NM II inhibitor (‐)002Dblebbistatin or MYH‐9 shRNA, the synergistic inhibition effect of DT‐13 and TPT on aerobic glycolysis was eliminated in BGC‐823 cells. Immunohistochemical analysis revealed selective up‐regulation of NM IIA while specific down‐regulation of p‐CREB, EGFR, and HK II by the combination therapy. Collectively, these findings suggested that this regimen has significant clinical implications, warranted further investigation.

## 1 INTRODUCTION

Gastric cancer (GC) is one of the most common cancers worldwide, especially in developing countries.^1^, ^2^ Despite improved surgical resection and efficient adjuvant therapy at the early stages of the disease, GC patients have a poor prognosis and low 5 year survival rate, with the frequent occurrence of ensuing relapse and metastasis.^3^ Moreover, advanced gastric cancers are resistant to traditional therapies and modern treatments are therefore failed.^4^, ^5^ Therefore, it is of great importance to develop novel therapies for gastric cancer.

Aerobic glycolysis was a process with the involvement of many enzymes, for example hexokinase II (HK II), phosphofructokinase‐1 (PFK‐1), pyruvate kinase M2 (PKM2) and lactate dehydrogenase (LDHA).^6^ Some of them have been reported to be overexpressed in tumours, including GC, and can be regulated by many oncoproteins to promote tumour proliferation, migration and chemoresistance.^7^ Accelerated glucose uptake during aerobic glycolysis and loss of regulation between glycolytic metabolism and respiration are the major metabolic changes found in malignant cells.^7^, ^8^ Interest in targeting cancer metabolism has been renewed in recent years, and more and more enzymes related to aerobic glycolysis were found to be as potential targets for drug treatment, including HK II, PKM2, glucose transporter 1 (GLUT1) and so on.^9^, ^10^ Metabolism and the aerobic glycolysis of cancer cells are seen as specific target of cancer cell, which have provided a new view for cancer treatment.^11^, ^12^

Topotecan, a semi‐synthetic analogue of the new Topo‐inhibitor camptothecin, has been licensed as an anticancer agent for small cell lung cancer (SCLC),^13^ ovarian cancer,^14^ head‐neck tumours, and gastrointestinal carcinoma. The usage of TPT on treating GC and SCLC is limited by its side‐effects such as toxicity and suffered from the potential drug resistance. DT‐13, a saponin of the dwarf lilyturf tuber *Ophiopogon japonicus* wall (Family: Convallariaceae), possesses anticancer activities against various types of cancers and antiangiogenesis activity 15 on multiple targets, such as early growth response 1 (Egr‐1), VEGF, CCR‐5, HIF‐1α and MMP2/9.^16^, ^17^, ^18^ To increase the efficacy of TPT and avoid the risk of resistance development, we designed combination therapies of DT‐13 and TPT and found that DT‐13 enhanced the pro‐apoptotic effect of TPT on GC by up‐regulating NM IIA/EGFR/Cav‐1 axis.^19^

As a key target of our combination therapies, NM IIA, encoded by MYH‐9, is an ATP‐driven molecular motor that plays diverse roles in cell physiological functions such as cell migration, adhesion, polarization and cytokinesis.^20^, ^21^, ^22^ NM IIA was essential for the endocytosis of EGFR and the modulation of the EGFR‐dependent activation of downstream signals, including ERK1/2 and AKT.^23^ In our previous research, we further confirmed the relationship of NM IIA and EGFR and that the ability of DT‐13 to enhance the pro‐apoptotic effect of TPT on GC via myosin IIA‐induced endocytosis of EGFR in vitro and in vivo.^19^ Meanwhile, it had been reported that EGFR signalling was also associated with increased glycolysis: activated the first step in glycolysis.^24^ On the contrary, EGFR inhibitors may reactivate oxidative phosphorylation of cancer cells and provide a mechanistic clue for the rational combination of agents targeting EGFR‐dependent proliferation and glucose metabolism in cancer therapy.^25^

Based on the finding that NM IIA induced the endocytosis of EGFR and the fact that EGFR could increase the activity of aerobic glycolysis, we developed the hypothesis that DT‐13 might be synergistically combined with TPT to inhibit aerobic glycolysis in high EGFR expression GCs through increasing NM IIA modulation of EGFR endocytosis and downstream signalling.

## 2 MATERIALS AND METHODS

### 2.1 In vivo tumorigenicity

Female athymic BALB/c nude mice (5‐6 weeks old) with body masses ranging from 18 to 20 g were supplied by the Shanghai Institute of Materia Medica, Chinese Academy of Sciences. BGC‐823 cells were collected in serum‐free medium (10^6^ cells/100 μL). Then, the cell suspension was injected subcutaneously into four mice in one flank as host. After 2 weeks, the resulting subcutaneous tumour (≈2 cm in diameter) were surgically removed under strict aseptic conditions following removal of necrotic tissue from the central tumour areas, and cut into small cubic fragments of approximately 1 mm^3^. Nude mice were randomly divided into two groups: 54 as tumour bearing mice were explanted with tumour fragments from the BGC‐823 cell lines, while the other six were assigned as sham operation group (SHAM). In brief, mice were anaesthetized by i.p. injection of sterile pentobarbital solution (50 mg/kg of body weight, China Academy of Military Medical Science). For implantation, the mouse stomach was gently exteriorized via a left‐side upper abdominal incision, and one small tissue pocket was prepared in the middle wall of the greater curvature using microscissors, and then, one tumour piece was placed into the pocket. The stomach was then returned to the peritoneal cavity, and the abdominal wall was closed with 4‐0 absorbable sutures. The mice were given special care and fed in cages as usual after surgery. After a week, 54 tumour‐bearing mice were divided into nine groups according to their weight (n = 6): BGC‐823 tumour‐bearing mice (BGC); BGC mice injected intravenously with 8.0 mg/Kg (HTPT) or 0.5 mg/Kg (LTPT) dissolved in saline twice a week; BGC mice administrated intragastrically with 2.5 mg/Kg (HDT), 1.25 mg/Kg (MDT) or 0.625 mg/Kg DT‐13 (LDT) suspended in 0.5% sodium carboxymethylcellulose (CMC‐Na) once a day; BGC mice combined administration group with 0.5 mg/Kg TPT and 2.5 mg/Kg (HD‐LT), 1.25 mg/Kg (MD‐LT) or 0.625 mg/Kg DT‐13 (LD‐LT). However, the mice in SHAM group were treated with normal saline. All nude mice were observed the growth and physical condition every day, weighed three times a week for 3 weeks. After 3 weeks of administration, the nude mice were killed, and the tumours were completely removed. Animal care and surgery protocols were approved by the Animal Care Committee of China Pharmaceutical University. All animals were treated appropriately and used in a scientifically valid and ethical manner.

### 2.2 Preparation of samples and acquisition of ^1^H NMR spectra

The deep‐frozen serum samples were thawed at 4°C overnight and then were vortexed to remove any precipitates. To 300 μL of each serum sample, 150 μL phosphate buffer was added, which was dissolved in D_2_O (0.2 M, Na_2_HPO_4_/NaH_2_PO_4_ and pH 7.0) containing 0.05% TSP‐d_4_ as chemical shift reference. Samples were vortexed and then centrifuged at 12 000 *g* for 10 minutes at 4°C to afford 500 μL of supernatant.

Frozen tissue sections were weighed (ca. 250 mg), homogenized in precooled acetonitrile/water (vol/vol = 1:1, 5 mL/g tissue) kept in an ice/water bath and centrifuged (12 000 *g*, 10 minutes, 4°C).^38^ The supernatant was lyophilized and then reconstituted in 600 mL phosphate buffer dissolved in D_2_O. After vortexing and centrifugation (12 000 *g*, 10 minutes, 4°C), a total of 550 mL of the supernatants was pipetted into 5 mm NMR tubes for analysis.

All the ^1^H NMR spectra were recorded at 298 K on a Bruker AV‐500 MHz spectrometer. The water‐suppressed Carr‐Purcell‐Meiboom‐Gill (CPMG) spin‐echo pulse sequence (RD‐90°‐ (τ‐180°‐τ) n‐ACQ) with a total spin‐echo delay (2nτ) of 40 ms was used to attenuate broad signals from proteins and lipoproteins. Typically, 128 transients were acquired with 32 K data points for each spectrum with a spectral width of 10 kHz.

### 2.3 NMR data processing and multivariate data analysis

With TopSpin software (version 3.0, Bruker Biospin), all the ^1^H NMR spectra were automatically corrected for phase and baseline distortions, and calibrated to TSP at 0.00 ppm. The spectral regions between δ 0.8 and 9.0 ppm for each tissue sample were automatically data reduced to integral segments using an adaptive binning approach based on the code 39 implemented in MATLAB (version 7.3, MathWorks). The area under the spectrum was then calculated for each segmented region and expressed as an integral value. The regions of water resonance (4.59‐5.15 ppm) were removed to avoid the effects of imperfect water suppression. To account for variations in concentration of metabolites due to dilute, the binned data were probabilistic quotient normalized using a median calculated spectrum,^40^ mean‐centred and pareto scaled in R, a freely available, open‐source software (R Development Core Team, http://cran.r-project.org/) prior to multivariate statistical analysis. A supervised orthogonal partial least‐squares discriminant analysis (OPLS‐DA) was performed using scripts written in R language.

### 2.4 Identification of metabolites

The statistical total correlation spectroscopy (STOCSY) technique was used to identify multiple NMR peaks from the same molecule in a complex mixture, which took advantage of the multicollinearity of the intensity variables in a set of spectra. Aided by STOCSY, metabolites were identified by comparing with those reported in the literature and/or registered in Human Metabolome Database (HMDB) (www.hmdb.ca). The assignments were further confirmed by 2D NMR techniques such as total correlation spectroscopy (TOCSY) and heteronuclear singular quantum correlation (HSQC).

### 2.5 Cell culture

Human gastric cancer BGC‐823 cells were purchased from The Shanghai Institute of Life Science, Chinese Academy of Sciences. The cells were maintained in DMEM with 10% foetal bovine serum (FBS, Gibco) and antibiotics (100 units/mL penicillin and 100 mg/mL streptomycin) and then were incubated in a humidified atmosphere with 5% CO_2_ at 37°C. Cells were grown to 80% confluence and treated with DT‐13 (10 μmol/L), TPT (0.1, 1 μmol/L) or DT‐13‐TPT combination treatment for 48 hours.

### 2.6 Cell transfection

The MYH‐9 lentivirus shRNA was purchased from Shanghai GenePharma Co., Ltd. The cells were seeded at a density of 5 × 10^4^/well in a 24‐well plate 24 hour before transfection to achieve more than 70% confluence. 20 μL MYH‐9 lentivirus shRNA and 20 μL scrambled sequence lentivirus shRNA were added into 2 mL fresh medium individually and then added 2 μL polybrene (Santa Cruz) after 24 hour treatment, and lentivirus medium was replaced by fresh medium. The plate was incubated at 37°C for 48‐72 hours until the transfection efficiency was more than 80% and was then used in the experiments described below. The sequence for MYH‐9 shRNA was forward, 5‐GAGGCAAUGAUCACUGACUdTdT‐3; reverse, 5‐AGUCAGUGAUCAUUGCCUCdTdT‐3.

### 2.7 Co‐immunoprecipitation assays

HK II (Santa Cruz) was immunocaptured from cells extracts using polyclonal antibodies to HK II cross‐linked to protein G‐agarose beads (Thermo Fisher Scientific). The immune complexes were analysed by Western blotting and probed with antibody against VDAC (Cell Signaling Technology).

### 2.8 Immunofluorescence assays

The cells were fixed with 4% paraformaldehyde in PBS at 30 minutes intervals, permeabilized with 0.5% Triton X‐100 and blocked with 3% BSA for 1 hour. Incubation with primary antibodies (diluted 1:200; Cell Signaling Technology) against p‐ERK, p‐AKT (diluted 1:50; Santa Cruz) against HK II and Mito‐Tracker Green FM (Invitrogen) was carried out overnight at 4°C. Second antibodies (Alexa Fluor^®^ 488 dye and Alexa Fluor^®^ 594 dye (Life Technologies)) were incubated 1 hour and then cover‐slipped with ProLong Gold antifade with DAPI (Life Technologies) 5 minutes before imaging. A laser scanning confocal microscope FV10‐ASW (Ver 2.1, MPE FV1000; Olympus Corp.) was used for imagining.

### 2.9 Immunohistochemical analysis for NM IIA, EGFR, p‐CREB and HK II in tumour tissues

Paraffin‐embedded tissue sections were deparaffinized in xylene followed by treatment with a graded series of alcohol [100%, 95% and 80% (pH 7.5). Antigen retrieval for ethanol/double‐distilled H_2_O (v/v)] and rehydrated in PBS paraffin‐embedded tissues was performed with sodium citrate 0.01 mol/L (pH 6.0) for 98°C for 5 minutes. Endogenous peroxidase was blocked by the use of 3% hydrogen peroxide in methanol for 10 min. The samples were washed thrice with PBS and incubated for 20 min at room temperature with a protein blocking solution containing 5% normal horse serum and 1% normal goat serum in PBS. Excess blocking solution was drained, and the samples were incubated overnight at 4°C with one of the following: 1:800 dilution of rabbit polyclonal anti‐human p‐CREB antibodies (Cell Signaling Technology), 1:50 dilution of anti‐human HK II antibodies (Santa Cruz), 1:100 dilution of anti‐human EGFR antibodies, and 1:50 dilution of anti‐human NM IIA antibodies. The samples were then rinsed thrice with PBS and incubated for 1 hour at room temperature with the appropriate dilution of the secondary antibody. Slides were examined under the microscope. The positive areas were analysed using by Image‐pro plus software analysis.

### 2.10 Western blot analysis

Cellular proteins were extracted, and Western blot analysis was performed as previously described after BGC‐823 cells were treated with DT‐13 and TPT for 48 hour. All primary antibodies were purchased from Cell Signaling Technology, Inc (Cell Signaling Technology). Horseradish peroxidase (HRP)–conjugated antimouse immunoglobulin G (Sigma‐Aldrich) and anti‐rabbit immunoglobulin G (Cell Signaling Technology) were used as the secondary antibodies. Protein bands were visualized using enhanced chemiluminescence reagents (Millipore).

### 2.11 Quantitative real‐time PCR

Total cellular RNA was isolated with the TRIzol^®^ Reagent (Vazyme) and reverse‐transcribed with the RevertAid^TM^ First Strand cDNA Synthesis Kit (Takara). The mRNA level was measured with the SYBR Green master mix (Vazyme). The amount of mRNA for each gene was standardized with the internal control (18s mRNA). Each treatment group was compared with the control group to show the relative mRNA level. The primer sequences for quantitative RT‐PCR are provided in Table S1.

### 2.12 Glucose, lactate, ROS, ATP detection

The levels of lactic acid, glucose, ROS, ATP in cells supernatant were collected after the combination treatment for 48 hours and then assayed following the manufacturer's instructions of the related kit.

### 2.13 Statistical analysis

All of the results excluding metabolomics analysis in vivo were presented as the mean ± SD from triplicate experiments performed in a parallel manner unless otherwise indicated. Statistically significant differences (one‐way ANOVAs followed by Bonferroni's multiple comparison test) were determined using GraphPad Prism 6 software. A value of *P* < 0.05 was considered significant, and values of *P* < 0.01 and *P* < 0.001 were considered highly significant. The *P*‐values of metabolomics analysis in vivo were corrected by BH (Benjamini‐Hochberg) methods and were calculated based on a parametric Student's *t* test or a nonparametric Mann‐Whitney test (dependent on the conformity to normal distribution), * *P* < 0.05, ** *P* < 0.01, *** *P* < 0.001.

## 3 RESULTS

### 3.1 DT‐13 combined with TPT had synergistically antitumour effect and aerobic glycolysis inhibition effect in BGC‐823 orthotopic xenograft nude mice

**Figure 1:**
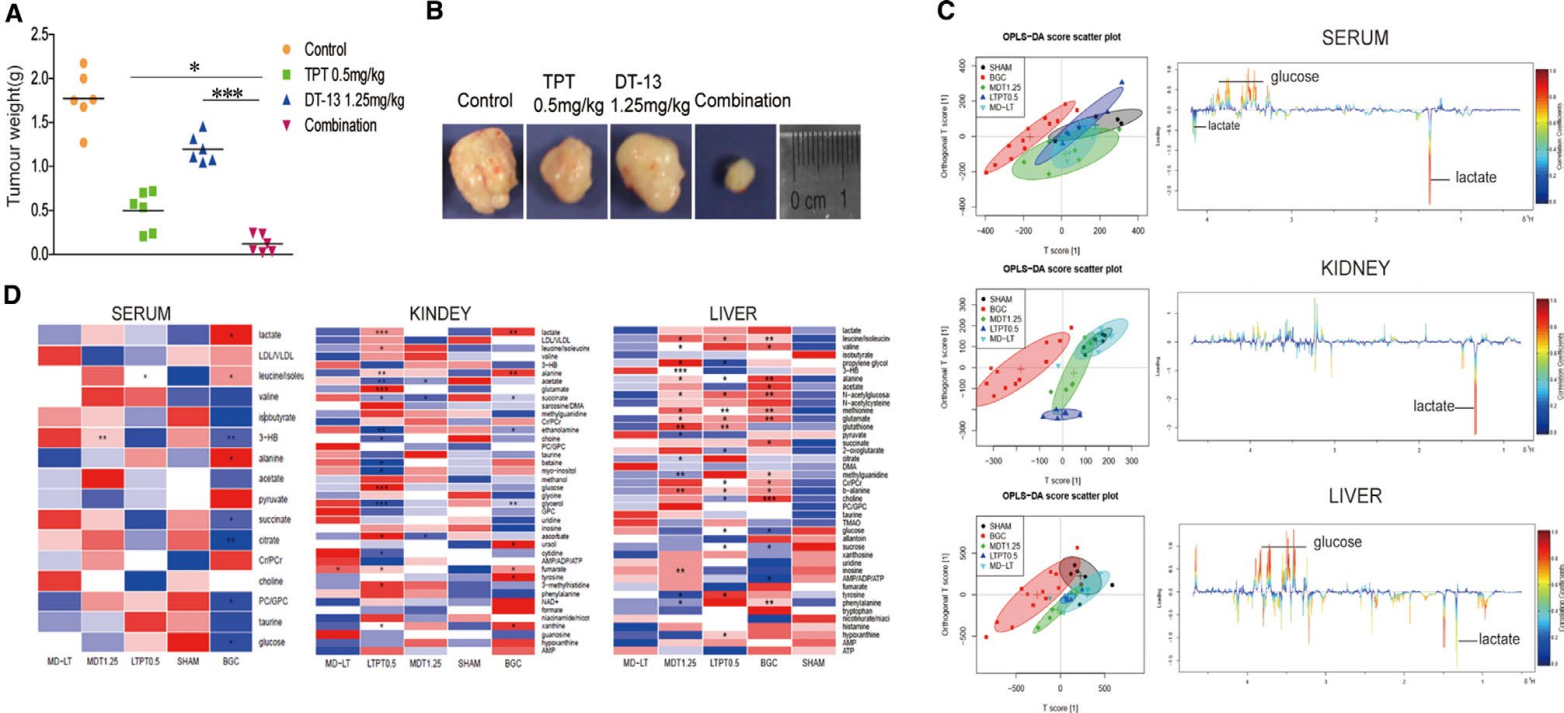
Combination treatment and inhibition of tumour growth in BGC‐823 xenograft nude mice, and metabolomics analysis in vivo. A, The weight of the tumours in nude mice was examined*.* B, Representative photographs of tumours, respectively, in each group at the end of experiment. C, Scores plot and colour‐coded loadings plot according to the OPLS‐DA analysis of NMR data from different tissue extracts of mice: serum; kidney; liver. Significantly changed metabolites were assigned in the loadings plots. Negative signals represent increased and positive signals represent decreased concentrations in model group. D, Potential marker metabolites in mice serum, kidney, liver identified by 1H‐NMR and their fold changes among groups and the associated *P*‐values. Colour‐coded according to the fold change value, red represents increased and blue represents decreased concentrations of metabolites. *P*‐values corrected by BH (Benjamini‐Hochberg) methods were calculated based on a parametric Student's *t* test or a nonparametric Mann‐Whitney test (dependent on the conformity to normal distribution). * *P* < 0.05, ** *P* < 0.01, *** *P* < 0.001

In our previous study, we had found that DT‐13 combined with TPT showed stronger antitumour effect in mice subcutaneous xenograft model in vivo compared with their individual effects.^19^ Here, we further evaluated the effect of DT‐13‐TPT combination in vivo using an established BGC‐823 cell orthotopic xenograft model. As shown in Figure 1A,B and Figure S1A, 1.25 mg/kg DT‐13 combined with 0.5 mg/kg TPT exerted a significantly synergistic inhibitory effect on BGC‐823 orthotopic xenografts.

To further investigate the therapeutic effects and excavate potential metabolic mechanism of the combination treatment on GC, orthogonal partial least‐squares discriminant (OPLS‐DA) analysis was performed on ^1^H NMR data of serum, spleens, kidneys, and livers, with the typical NMR spectrum (Figure S1B,C,D). In the OPLS‐DA scores plots, BGC‐823 bearing model group and sham group were wholly or partly separated (Figure 1C), indicating the metabolic disorder in BGC‐823 xenograft model. The combination treatment group was severely overlapped with the sham group and was the furthest away from the model group in the OPLS‐DA scores plots, suggesting exceptional good remedial effect of DT‐13‐TPT combination treatment on GC among others. The colour‐coded loadings plots revealed significant difference in the metabolites between the combination treatment group and model group (Figure 1C). The combination treatment group showed significantly lowered levels of lactate, pyruvate, alanine, glutamate, creatine/creatine phosphate (Cr/PCr), leucine, AMP, tyrosine, uracil, nicotinamide adenine dinucleotide (NAD+), and notably elevated levels of 3‐hydroxybutyric acid (3‐HB), succinate, citrate, choline, glucose, taurine and uridine than individual treatment groups (Figure 1D). It could be concluded that the combination treatment group could more effectively reverse the abnormal metabolic status towards a normal condition.

### 3.2 The combination treatment synergistically inhibited the expression of the aerobic glycolysis‐related enzymes in vivo and in vitro

**Figure 2:**
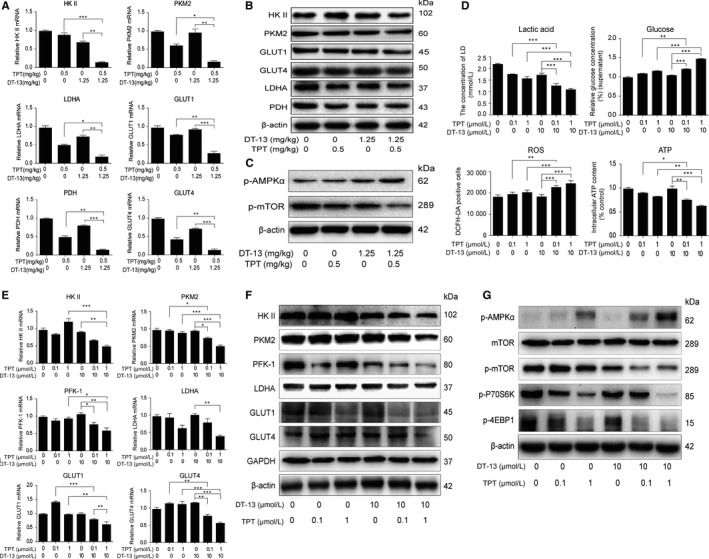
DT‐13 combined with TPT promoted aerobic glycolysis inhibition effects in BGC‐823 cells in vivo and in vitro by up‐regulating the activity of AMPKα. (A‐B) Effects of DT‐13 combined with TPT on the mRNA levels A, and protein levels B, of the aerobic glycolysis‐related enzymes in BGC‐823 tumour tissue. C, The AMPK‐mTOR pathway was detected using Western blot analysis when treated with DT‐13 combined with TPT in BGC‐823 tumour tissue. D, The combination inhibitory effects of DT‐13 combined with TPT on the levels of lactate, ATP, glucose and ROS in BGC‐823 cells were measured after the combined treatment for 48 hours. The aerobic glycolysis‐related enzymes were assessed by PCR and Western blot analysis in BGC‐823 cells (E‐F). G, After the cells were treated with the combined treatment for 48 hours, the AMPK‐mTOR pathway was assessed by Western blotting analysis in BGC‐823 cells. Statistical analysis was performed using one‐way ANOVA followed by Bonferroni's multiple comparison test, *P < 0.05; **P < 0.01; ***P < 0.001; for A, D and E, statistical analysis was performed using at least three independent replicates

Metabolomics analysis in vivo revealed the combination treatment had stronger inhibition effect on glucose uptake and lactate production than the individual treatment effects, which indicate that the combination treatment might synergistically inhibit the aerobic glycolysis. In tumour tissues from the BGC‐823‐xenografted nude mice treated with DT‐13‐TPT combination, the expressions of aerobic glycolysis‐related enzymes (HK II, PKM2, LDHA, and so on) were inhibited (Figure 2A,B). Treated with the combination therapy (10 μmol/L DT‐13 + 0.1 μmol/L TPT; 10 μmol/L DT‐13 + 1 μ μmol/L TPT) for 48 hour in vitro, BGC‐823 cells were observed with decreased glucose uptake, lactate production, ATP production and reactive oxygen species (ROS) consumption (Figure 2D). In addition, the combination treatment also exhibited a stronger inhibitory effect on the expression of aerobic glycolysis‐related enzymes than TPT or DT‐13 alone in BGC‐823 cells (Figure 2E,F), which were consistent with the results in vivo.

Due to the key role of the AMPK signalling in down‐regulating tumour energy metabolism,^26^ we next investigated the effect of the combination treatment on the AMPK‐mTOR pathway. We observed that the combination treatment increased the p‐AMPKα level and inhibited the activity of mTOR pathway in vivo and in vitro (Figure 2C,G). These results indicated that DT‐13 combined with TPT exerts an aerobic glycolysis inhibition activity via activating AMPK and inhibited its downstream mTOR pathway in vivo and in vitro.

### 3.3 Combinational aerobic glycolysis inhibition effect was promoted by NM IIA

As we found the inhibitory of combination treatment on aerobic glycolysis in vivo and in vitro, we desired to find out the potential mechanism. According to the finding that DT‐13 enhanced the pro‐apoptotic effect of TPT on GC via myosin IIA‐induced endocytosis of EGFR in vitro and in vivo,^19^ we wondered whether the combinational aerobic glycolysis inhibition effect was correlated to NM IIA. We first examined the relationship of NM IIA and aerobic glycolysis. In NM IIA knock‐downed BGC‐823 cells, the mRNA levels of HK II, PKM2, PFK‐1 and LDHA were all increased, and only HK II protein level was increased markedly (Figure S2A,B). Meanwhile, NM IIA knock‐down could increase the lactate production and glucose uptake (Figure S2C).

**Figure 3:**
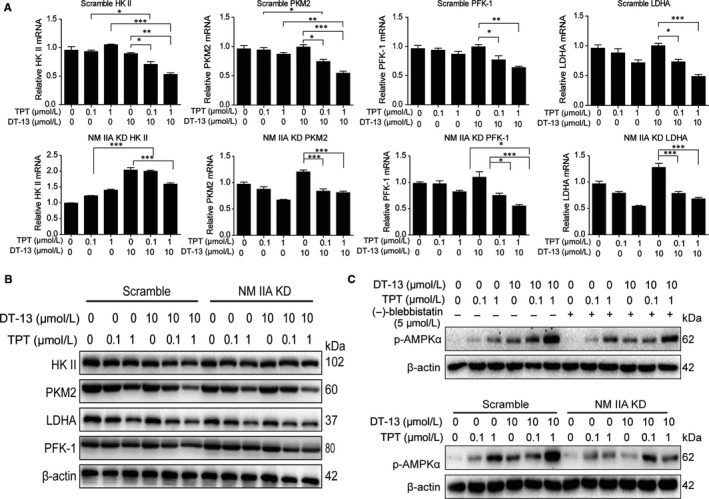
Combinational aerobic glycolysis inhibition effect was promoted by NM IIA. The cells were measured after the combined treatment for 48 hour. (A‐B) After NM IIA knock‐down, the expressions of aerobic glycolysis‐related enzymes were assessed by PCR and Western blot analysis in BGC‐823 cells. C, The cells were pre‐treated with NM II inhibitor (‐)‐blebbistatin or knocked‐down NM IIA; the effects of DT‐13 combined with TPT on AMPKα phosphorylated protein levels were detected by Western blot analysis in BGC‐823 cells. Statistical analysis was performed using one‐way ANOVA followed by Bonferroni's multiple comparison test, *P < 0.05; **P < 0.01; ***P < 0.001; for a, statistical analysis was performed using at least three independent replicates

To further test whether the combinational aerobic glycolysis inhibition effect was correlated to NM IIA, we next detected the change of the activities of the enzymes in NM IIA knock‐downed BGC‐823 cells treated with DT‐13‐TPT combination. We found that in NM IIA knock‐downed cell, the combinational treatment inhibition effect was reversed totally on HK II mRNA level; reversed a bit on PKM2 mRNA level; but could not reverse little on PFK‐1 and LDHA mRNA levels (Figure 3A). What's more, knocking‐down NM IIA could only reverse the combination treatment inhibition effect on HK II protein level in BGC‐823 cells (Figure 3B). The combination treatment promotion effect on ROS level was reversed in NM IIA knocking‐down cell (Figure S3A). We also found that knocking‐down NM IIA and (‐)‐blebbistatin could both reverse the activation of AMPK induced by the combination treatment (Figure 3C). When pre‐treated with APMK inhibitor dorsomorphin or ROS scavenger NAC for 2 hours and then treated with the combinational treatment for 48 hours, the decrease of HK II protein in BGC‐823 cells induced by the combinational treatment could not be reversed by dorsomorphin or NAC (Figure S3B,C). These findings showed that the change of HK II happened before the AMPK activation and ROS level increase; we speculated that DT‐13 combined with TPT increased the aerobic glycolysis inhibition activity through myosin IIA‐induced inactivation of HK II.

### 3.4 Effects of EGFR pathway on HK II in BGC‐823 cells

**Figure 4:**
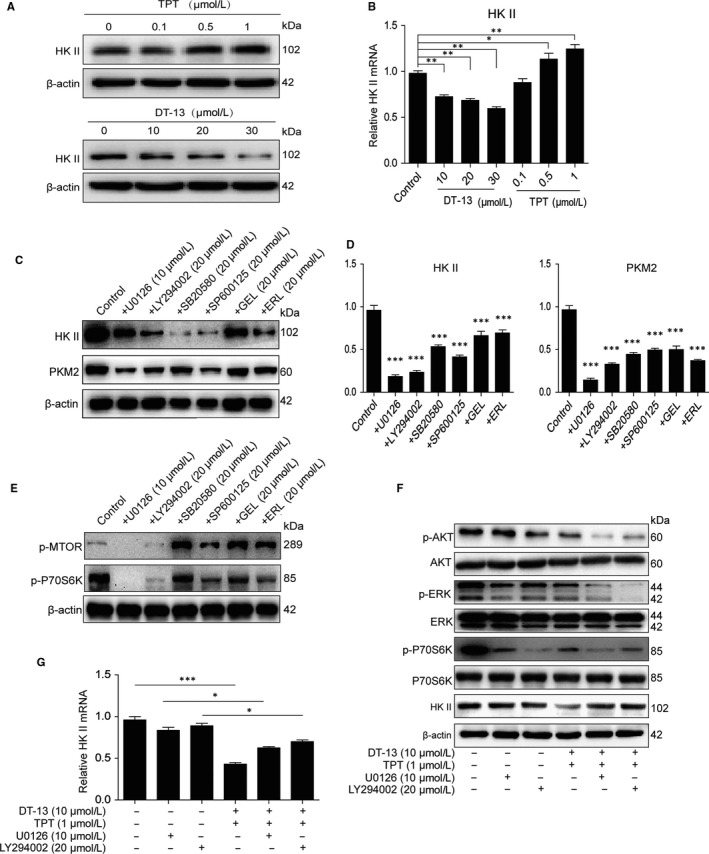
Effects of EGFR pathway on HK II. (A‐B) The effect of DT‐13 and TPT separately on HK II in BGC‐823 cells. The cells were treated with DT‐13 (10, 20, 30 μmol/L) or TPT (0.1, 0.5, 1 μmol/L) for 48 hour; the total protein level and mRNA level of HK II were checked by Western blot analysis. (C‐D) The HK II and PKM2 levels were detected using Western blot and PCR analysis when treated with the EGFR inhibitors and EGFR downstream inhibitors in BGC‐823 cells. E, Western blot assays were used to examine the effect of the EGFR inhibitors and EGFR downstream inhibitors on the activity of mTOR pathway in BGC‐823 cells. (F‐G) BGC‐823 cells were treated with LY294002 (20 μmol/L) or U0126 (10 μmol/L) separately for 2 hour before DT‐13‐TPT combination treatment, the HK II level was determined by Western blot and PCR analysis, and the total/phospho AKT, total/phospho S6K and total/phospho ERK1/2 were detected by Western blot analysis. Statistical analysis was performed using one‐way ANOVA followed by Bonferroni's multiple comparison test, *P < 0.05; **P < 0.01; ***P < 0.001; for b, d and g, statistical analysis was performed using at least three independent replicates

To verify our conjecture that DT‐13 combined with TPT increased the aerobic glycolysis inhibition activity through myosin IIA‐induced inactivation of HK II, we examined the effect of DT‐13 and TPT on HK II in BGC‐823 cells, respectively. We found that the expression of HK II was suppressed by DT‐13, whereas TPT increased the expression of HK II in BGC‐823 (Figure 4A,B). DT‐13 combined with TPT induced the endocytosis of EGFR by up‐regulation of NM IIA (Figure S3D), and EGFR pathway was correlated to aerobic glycolysis.^25^ We next examined the effects of EGFR pathway on the activity of HK II and PKM2. As shown in Figure 4C,4, the inhibitors of EGFR (erlotinib, gefitinib) and its downstream (U0126 (MEK inhibitor), LY294002 (PI3K inhibitor), SB20580 (p38 inhibitor), SP600125 (JNK inhibitor)) all decreased the protein and mRNA levels of HK II and PKM2 in BGC‐823 cells. Meanwhile, we also found that only two (LY294002 and U0126) out of the six inhibitors could inhibit the activity of mTOR pathway in BGC‐823 cells (Figure 4E). To further confirm the key role of MEK, PI3K pathways in the process of EGFR regulatory HK II, BGC‐823 cells were treated with 20 μmol/L LY294002 or 10 μmol/L U0126 separately for 2 hour before DT‐13‐TPT combination treatment. The combination treatment inhibition effect on the protein and mRNA levels of HK II was reversed (Figure 4F,G). This finding suggested that DT‐13 combined with TPT inhibited EGFR downstream MEK, PI3K pathway to suppress HK II, thereby inhibiting AMPK‐mTOR pathway, ultimately leading to the inhibition aerobic glycolysis in BGC‐823 cells.

### 3.5 DT‐13 combined with TPT inhibited p‐CREB by down‐regulating the activity of EGFR downstream pathways

**Figure 5:**
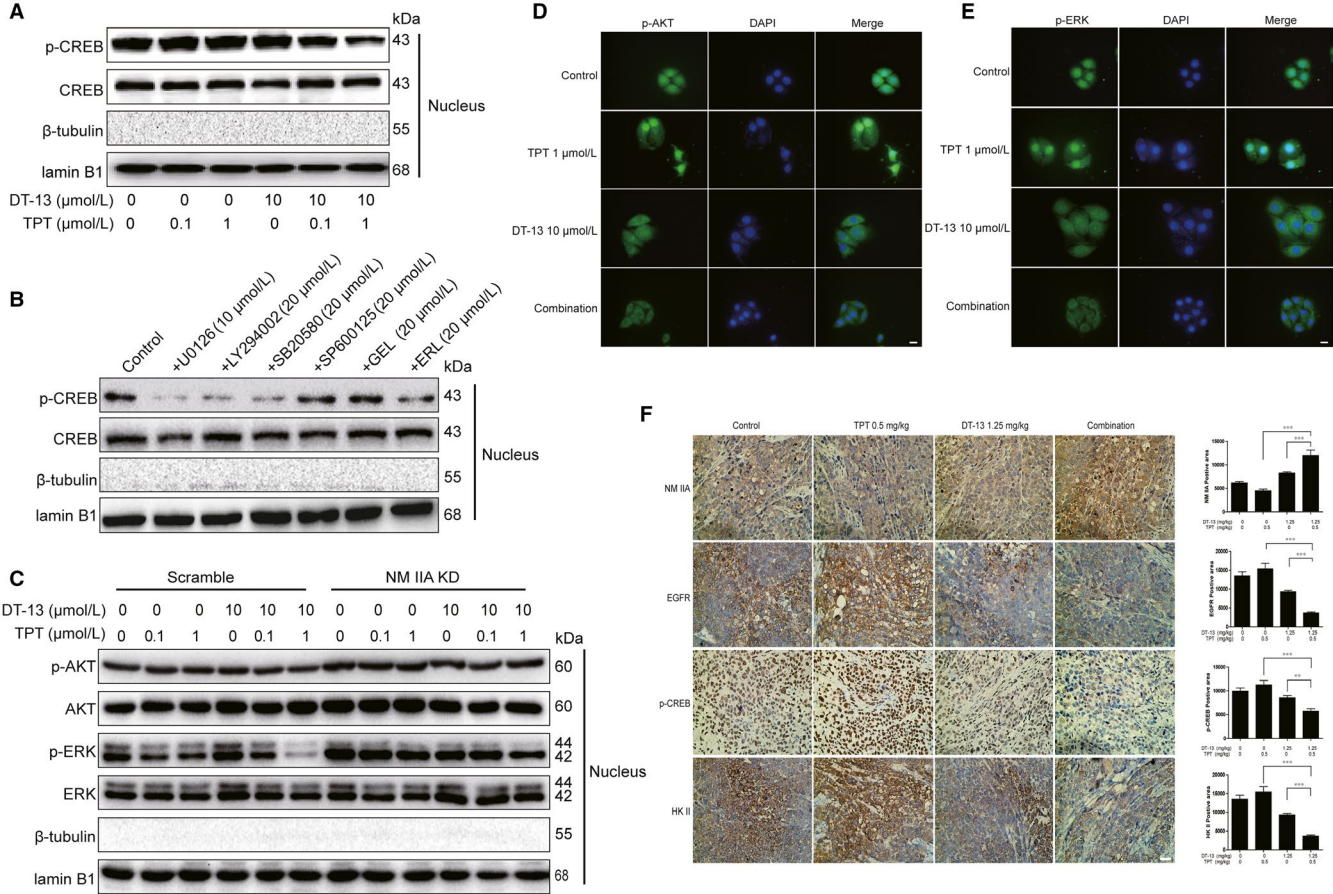
DT‐13 combined with TPT could inhibit p‐CREB by down‐regulating the activity of EGFR downstream pathways in vivo and in vitro. A, The CREB and p‐CREB levels in cell nucleus were detected using Western blot analysis when treated with DT‐13 combined with TPT for 48 hours in BGC‐823. B, The CREB and p‐CREB levels in cell nucleus were detected using Western blot analysis when treated with the EGFR inhibitors and the EGFR downstream inhibitors in BGC‐823 cells. C, The total/phospho AKT and total/phospho ERK1/2 levels in cell nucleus were detected using Western blot analysis when treated with DT‐13 combined with TPT in BGC‐823 cells and NM IIA knock‐down BGC‐823 cells. (D‐E) The p‐ERK and p‐AKT levels in cell nucleus were detected using cell fluorescence analysis when treated with DT‐13 combined with TPT in BGC‐823 cells. F, The NM IIA, EGFR, p‐CREB, HK II were detected using immunohistochemical analysis when treated with DT‐13 combined with TPT in BGC‐823 tumour tissue. Statistical analysis was performed using one‐way ANOVA followed by Bonferroni's multiple comparison test, **P < 0.01; ***P < 0.001; for F, statistical analysis was performed using at least three independent replicates

The above data showed that DT‐13 combined with TPT inhibited both protein and mRNA level of HK II (Figure 2E,F). Thus, we speculated that the combination treatment inhibited the transcription process of HK II. cAMP response element binding protein (CREB) was transcription factor, which could bind to the promoter regions of HK II and induced HK II activation.^27^, ^28^, ^29^ What's more, CREB was correlated to NM IIB.^30^ Thus, we next examined the effect of the combination treatment on the activity of CREB in cell nucleus. We found that the combination treatment exhibited a stronger inhibitory effect on p‐CREB level than TPT or DT‐13 alone in BGC‐823 cells (Figure 5A).

CREB phosphorylation is mainly controlled by protein kinase A (PKA) and calcium‐dependent protein kinase (PKC), casein kinase (CK II), MAPK, AKT pathway.^31^, ^32^ Next, we further confirmed the effect of EGFR downstream MEK, PI3K pathway on p‐CREB level in cell nucleus by using the EGFR and its downstream inhibitors. Except for gefitinib, six inhibitors could inhibit the p‐CREB level in cell nucleus (Figure 5B). Then, we examined the effect of the combination treatment on the p‐AKT and p‐ERK levels in cell nucleus by using Western blot analysis (Figure 5C) and cell fluorescence analysis (Figure 5D,E). We observed that the combination treatment exhibited a stronger inhibitory effect on p‐AKT and p‐ERK protein level and fluorescence intensity than TPT or DT‐13 alone in cell nucleus. These results confirmed that the combination of DT‐13 with TPT inhibited the entrance of p‐ERK, p‐AKT into the cell nucleus, thereby inhibiting the activity of CREB.

### 3.6 Effect of NM IIA on the activity of CREB in BGC‐823 cells

We have confirmed that aerobic glycolysis was correlated to NM IIA (Figure S2A,B,C) and that DT‐13 combined with TPT suppressed HK II by up‐regulating NM IIA (Figure 3A,B). To further investigate the effect of NM IIA on HK II promoter activity, we examined the p‐CREB, p‐AKT, p‐ERK levels in the cell nucleus of NM IIA knock‐downed BGC‐823 cells, where the p‐CREB, p‐AKT and p‐ERK levels were all increased (Figure S4A). By using cell fluorescence analysis, we also found the fluorescence intensity of p‐AKT and p‐ERK was increased in the cell nucleus of BGC‐823 cells with NM IIA knock‐downed or treated with (‐)‐blebbistatin (Figure S4B,C). We also examined the p‐AKT and p‐ERK levels in the nucleus of NM IIA knock‐down cells treated with DT‐13‐TPT combination. We found that NM IIA knock‐down reversed the inhibition effect of the combinational treatment on p‐AKT and p‐ERK protein level in the cell nucleus (Figure 5F). In tumour tissues, the positive areas for NM IIA were increased, while the positive areas for p‐CREB, EGFR and HK II were reduced (Figure 5G). These results demonstrated that DT‐13 combined with TPT inhibited the entrance of p‐ERK, p‐AKT into the nucleus by up‐regulating NM IIA, thereby inhibiting CREB activity, and ultimately inhibiting HK II promoter activity.

### 3.7 Effects of the combination treatment on the binding of HK II to the mitochondria

**Figure 6:**
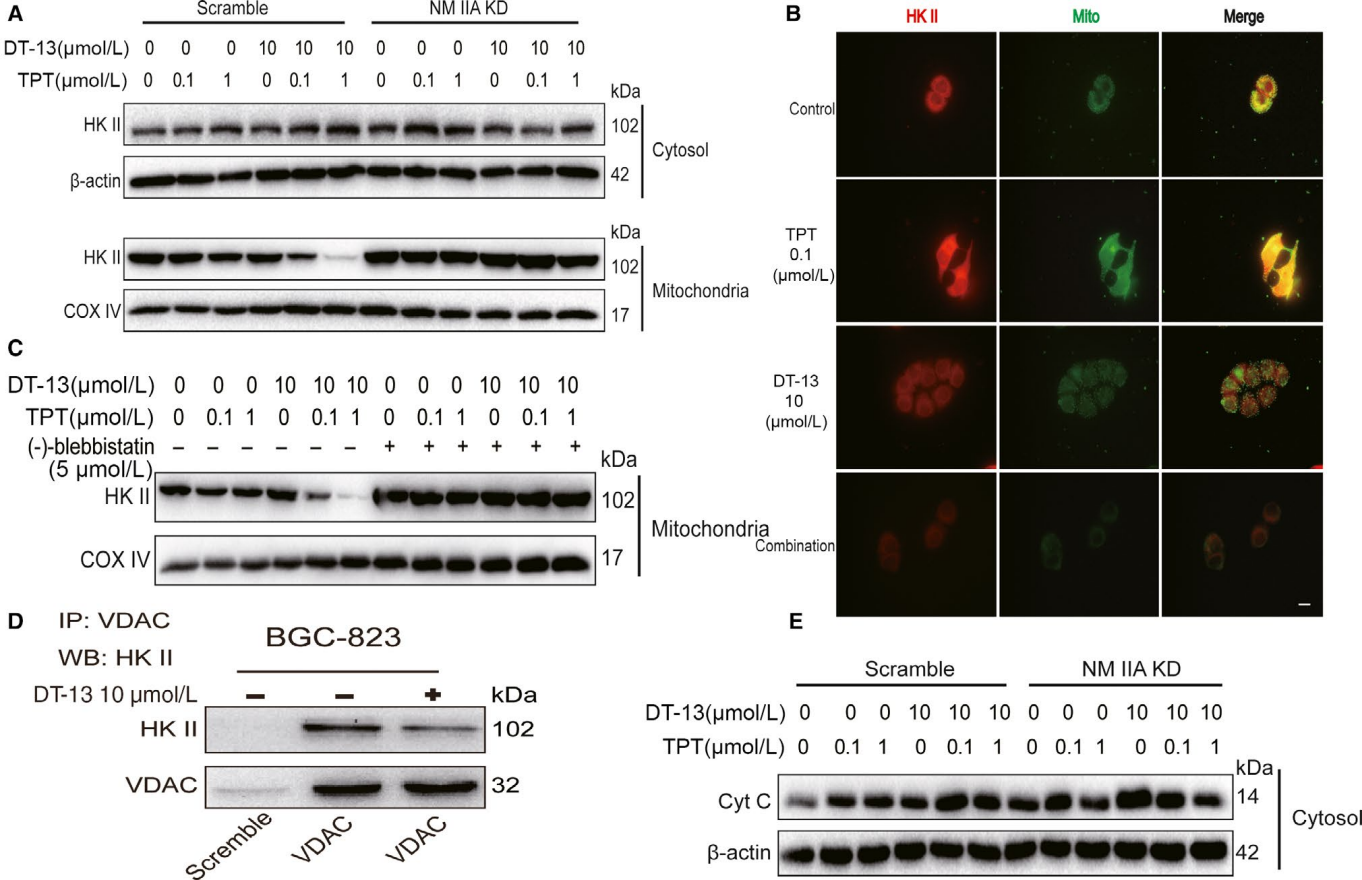
Effects of the combination treatment on the binding of HK II to the mitochondria. A, The HK II level in cytosol and mitochondria was detected using Western blot analysis when treated with DT‐13 combined with TPT in BGC‐823 cells and NM IIA knock‐down BGC‐823 cells. B, The immunofluorescence localization method was used to verify the binding of HK II to the mitochondria in BGC‐823 cells. The location of the arrowed line is to measure the position of the fluorescence intensity. C, The HK II level in mitochondria was detected when pre‐treated with (‐)‐blebbistatin for 2 hours before DT‐13 combined with TPT in BGC‐823 cells. D, The co‐immunoprecipitation assay was used to verify the interaction of VDAC with HK II in BGC‐823 cells. E, The cytochrome C level was detected using Western blot analysis when treated with DT‐13 combined with TPT in BGC‐823 cells and in NM IIA knock‐down BGC‐823 cells

The binding of HK II to mitochondria can combine aerobic glycolysis and oxidative phosphorylation together, not only make the hexokinase greater use of ATP, but also accelerate the glucose metabolism, providing more ADP for mitochondria and accelerate the tricarboxylic acid (TCA) cycle.^33^, ^34^ We found that the combinational treatment decreased HK II expression in mitochondria and increased HK II expression in cytosol (Figure 6A). By using ImageJ software analysis, we also found the combinational treatment decreased the co‐localization of HK II and mitochondria (Figure 6B). To further confirm the key role of NM IIA in inhibiting the binding of HK II to mitochondria by the combinational treatment, we examined the change of the HK II level in mitochondria and in cytosol when treated with DT‐13‐TPT combination by using NM IIA knock‐down cells or pre‐treated with (‐)‐blebbistatin in BGC‐823 cells. We found that both (‐)‐blebbistatin treatment and NM IIA knock‐down reversed the inhibition effect of combinational treatment on the binding of HK II to mitochondria (Figure 6C,D).

HK II is the only kind of glycolysis enzyme that can bind to mitochondria, whose binding sites are on the mitochondrial outer membrane protein (VDAC) channel. Once bound, it will inhibit mitochondrial release of cytochrome C (Cyt C) and tumour cell apoptosis.^33^ We used the co‐immunoprecipitation assay to determine the effect of the combinational treatment on the binding of HK II to VDAC. The results showed that the combinational treatment inhibited the binding of HK II to VDAC (Figure 6E). Meanwhile, the combinational treatment could promote mitochondrial release of Cyt C, which would be reversed in NM IIA knock‐downed BGC‐823 cells (Figure 6F). These results demonstrated that DT‐13 combined with TPT inhibited the binding of HK II to VDAC by up‐regulating NM IIA, thereby inhibiting aerobic glycolysis activity.

## 4 DISCUSSION

**Figure 7:**
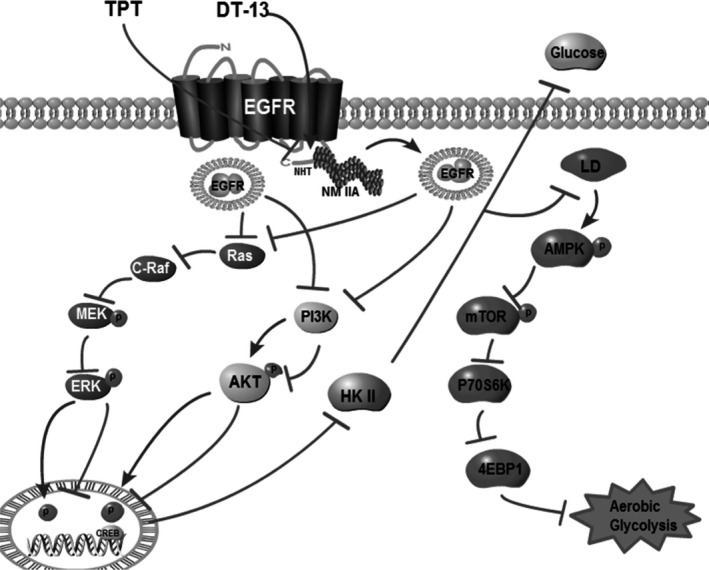
A schematic representation of a hypothesized mechanism of the synergistic combination of DT‐13 and TPT inhibits human gastric cancer aerobic glycolysis via NM IIA/EGFR/HK II axis

Overall, DT‐13 combined with TPT not only have synergistically pro‐apoptotic effect on BGC‐823 cells (high EGFR expression), but also synergistically inhibited the activity of the aerobic glycolysis in vitro and in vivo*.* Knock‐down of NM IIA in BGC‐823 cells reversed the synergistically aerobic glycolysis inhibition effect of the combination treatment (Figure 3 and Figure S3A), the inhibition effect of the combination treatment on the activity of HK II (Figures 3A,B,4F) and the inhibition effect of the combination treatment on the binding of HK II to mitochondria was reversed (Figure 6C,F). Upon further investigation, we found that with TPT, DT‐13 promoted EGFR ubiquitin‐mediated degradation through myosin IIA‐induced endocytosis of EGFR, further inhibited the activity of HK II by decreasing the transportation of phosphorylated forms of ERK 1/2 and p‐AKT into the nuclear and binding to CREB, then activated the AMPK pathway and eventually promoted the aerobic glycolysis inhibition activity more efficiently than with TPT or DT‐13 monotherapy. Moreover, DT‐13 combined with TPT could also inhibit the specific binding of HK II to mitochondria (Figure 7).

Potential marker metabolites in mice serum, spleen, kidney, liver identified by ^1^H‐NMR analysis were concerned with TCA cycle, branched chain amino acid (BCAA) metabolism, glutamine and glutamate metabolism, oxidative stress and aerobic glycolysis. For instance, succinate, an intermediate of the TCA cycle, was increased in the combination treatment group, which indicated the combination treatment might increase the TCA cycle.

In recent years, many cancer‐related pathways have found to be of profound effects on metabolism of cancer and oncogenic metabolic lesions may be selective targets for new anticancer therapeutics.^8^, ^12^, ^35^ Renewed interest in the fact that cancer cells have to reprogramme their metabolism in order to proliferate or resist treatment. It must take into consideration that the ability of tumour cells to adapt their metabolism to the local microenvironment (low oxygen, low nutrients).^36^ This variety of metabolic sources might be either a strength, resulting in infinite possibilities for adaptation and increased ability to resist chemotherapy‐induced death, or a weakness that could be targeted to kill cancer cells.^7^

In this study, we found that DT‐13‐TPT combination could inhibit the aerobic glycolysis‐related enzymes' activity and inhibit the binding of HK II to mitochondria, which have more effectively reverse the abnormal metabolic status towards a normal condition. Moreover, DT‐13 inhibited HIF‐1α expression in our previous study,^15^ and the HIF‐1α expression was strongly correlated with glycolysis.^37^ What's more, DT‐13 combined with TPT inhibited the binding of HK II to VDAC, inducing the increase of Cyt C release. Hence, whether the aerobic glycolysis inhibition effect of the combination treatment was also induced by HIF‐1α inhibition, and the fully suppression of aerobic glycolysis was the cause which induced of the mitochondrial apoptosis may all need to be explored in depth in the future.

The requirement of NM IIA for EGFR endocytosis was first reported by Kim JH.et al 23 and not further explored in depth and the binding of NM IIA and EGFR had not been applied to drug research until now. In our previous studies, DT‐13 had the ability to increase NM IIA expression and might promote NM IIA binding to EGFR, thereby expediting the endocytosis of EGFR.^19^ Our present study verified DT‐13 combined with TPT could inhibit the activity of HK II based on the interaction between NM IIA and EGFR, which efficiently, indicating the potential of this novel mechanism as a target for drug development.

In our previous study, we have reported NM IIA was related to cell apoptosis 19; however, the correlation between NM IIA and cell aerobic glycolysis has not been reported previously. Our findings highlight for the first time this correlation of NM IIA with cancer cell aerobic glycolysis by using MYH‐9 shRNA. These findings showed that NM IIA knocking down could increase lactate production and glucose uptake, and increase the activity of HK II and PKM2 (Figure S2). According to the structure of NM IIA and our present data (Figure S4), we also postulated that NM IIA might directly inhibit the activity of HK II in an EGFR independently way. Therefore, our research group will be investigating the whole picture of the contextual pathways between the NM IIA‐mediated HK II inactivation process.

## CONFLICT OF INTEREST

No potential conflicts of interests were disclosed.

## AUTHOR CONTRIBUTIONS

XY, DW, and LS designed the study. XY, DW, HD, YG and RL performed experiments. XY, DW, JW, SY and LS analysed the data. XY, YB, CQ, XL and JW interpreted the results. XY, DW and LS wrote the manuscript.

## Data Availability

The data that support the findings of this study are available from the corresponding author upon reasonable request.
