# Supplementary material for: Synergistic combination of DT‐13 and Topotecan inhibits aerobic glycolysis in human gastric carcinoma BGC‐823 cells via NM IIA/EGFR/HK II axis
Source: J Cell Mol Med. 2019 Aug 9;23(10):6622–34. doi: 10.1111/jcmm.14523 (PMC6787456; doi:10.1111/jcmm.14523)
Supplement: Supplementary file 1 [file JCMM-23-6622-s001.docx]

**Supplementary Material**

**
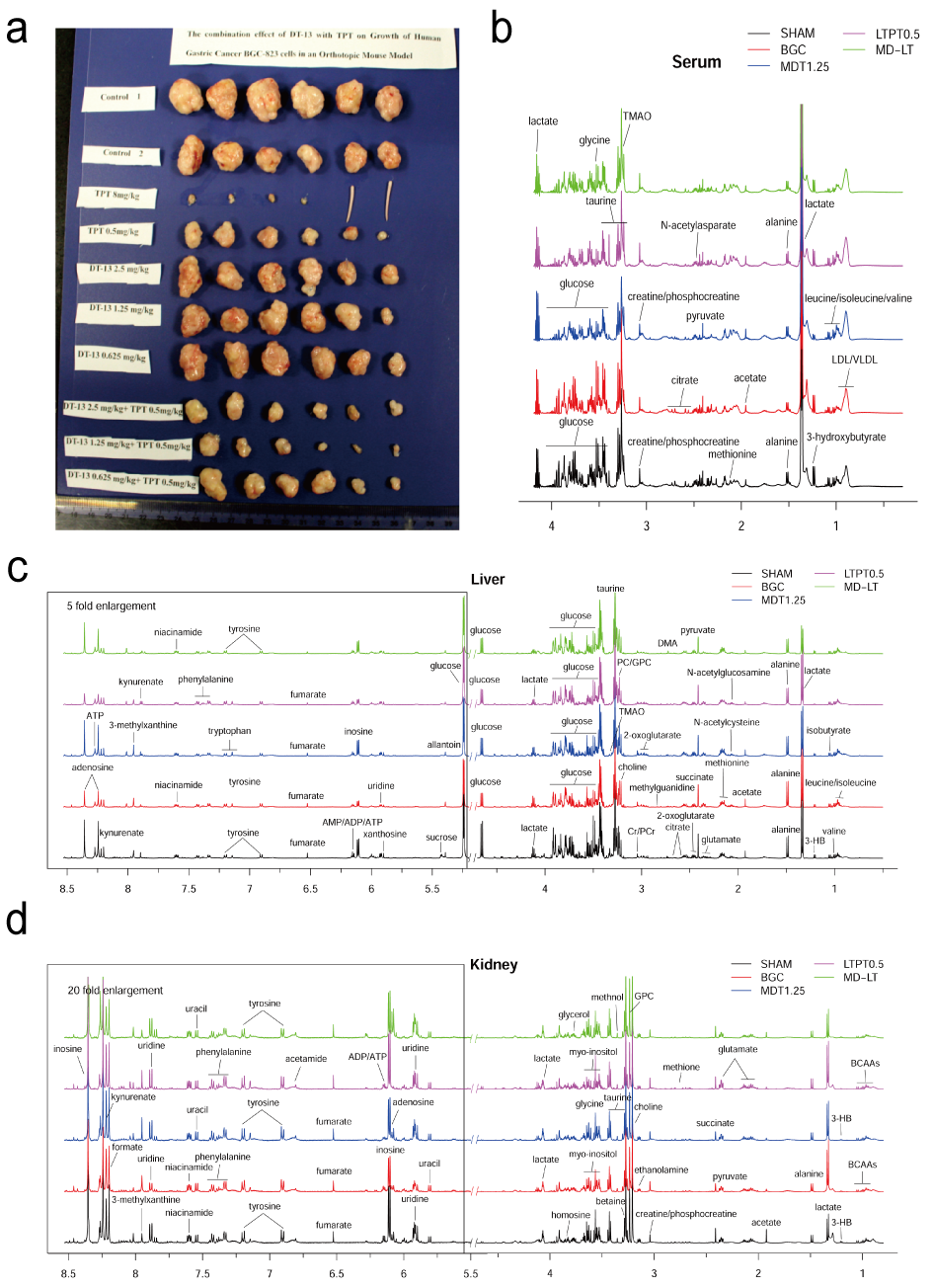
**

**Supplementary Figure S1: Typical 500 MHz ^1^H NMR spectra with the metabolites labeled.** (a) The tumors photographs of BGC-823 cell xenograft model. (b) Typical 500 MHz ^1^H NMR spectra of serum with the metabolites labeled. (c) Typical 500 MHz ^1^H NMR spectra of liver with the metabolites labeled. (d) Typical 500 MHz 1H NMR spectra of kidney with the metabolites labeled.


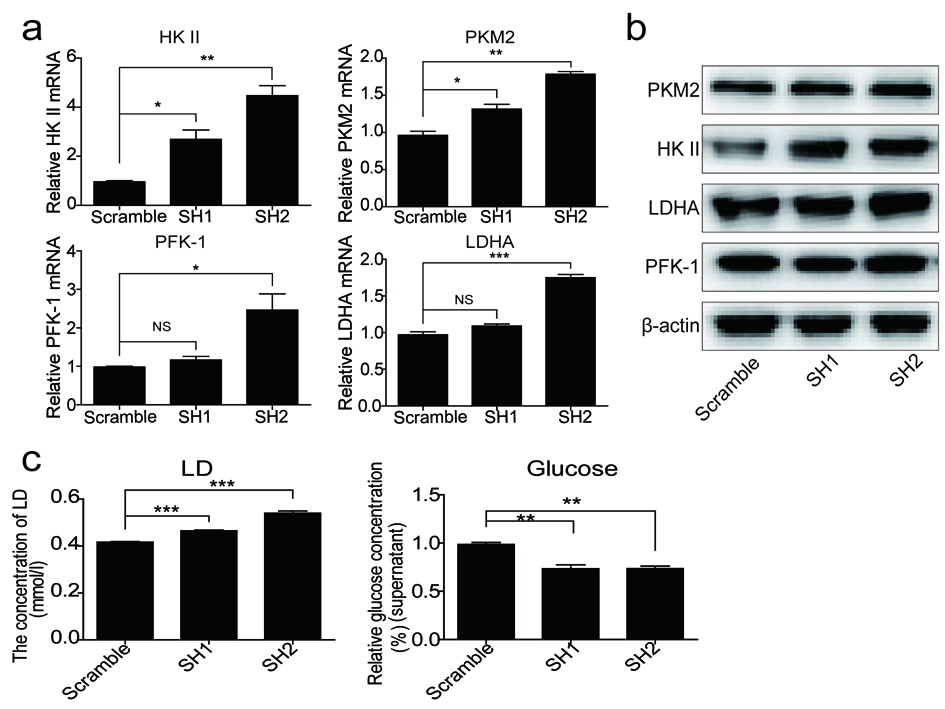


**Supplementary Figure S2: The correlation between NM IIA and aerobic glycolysis.** (a-b) PCR analysis and western blot analysis were using to detect the activity of the aerobic glycolysis related enzymes in BGC-823 NM IIA knock down cells. (c) The lactate generation and glucose uptake were detected in BGC-823 NM IIA knock down cells. Statistical analysis was performed using one-way ANOVA followed by Bonferroni's Multiple Comparison Test, *P< 0.05; **P< 0.01; ***P< 0.001; for a, c statistical analysis was performed using at least three independent replicates.


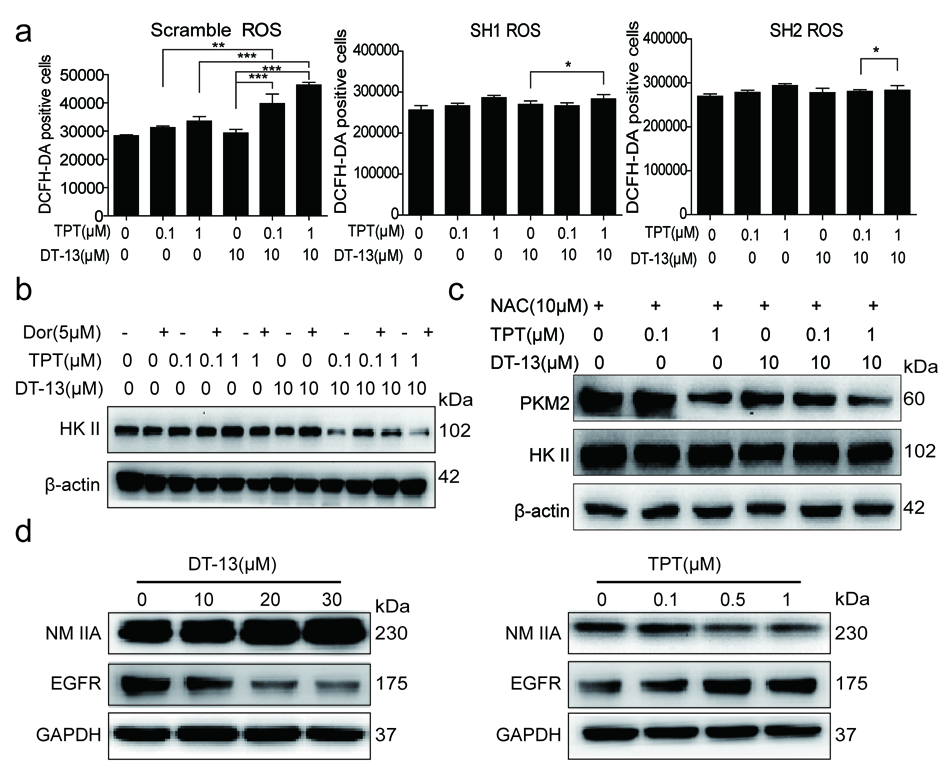


**Supplementary Figure S3: The relationship of NM IIA/EGFR/HK II in BGC-823 cells.** (a) The ROS level was detected after treatment with DT-13 and TPT for 48 h in BGC-823 NM IIA knock down cells. (b-c) The HK II level was detected using western blot analysis when pre-treated with Dorsomorphin (b) or NAC (c) for 2 h before DT-13 and TPT in BGC-823 cells. (d) The NM IIA and EGFR levels were detected using western blot analysis when cells were treated with DT-13 (10, 20, 30μM) or TPT (0.1, 0.5, 1 μM) alone for 48 h. Statistical analysis was performed using one-way ANOVA followed by Bonferroni's Multiple Comparison Test, *P< 0.05; **P< 0.01;***P< 0.001; for a statistical analysis was performed using at least three independent replicates.


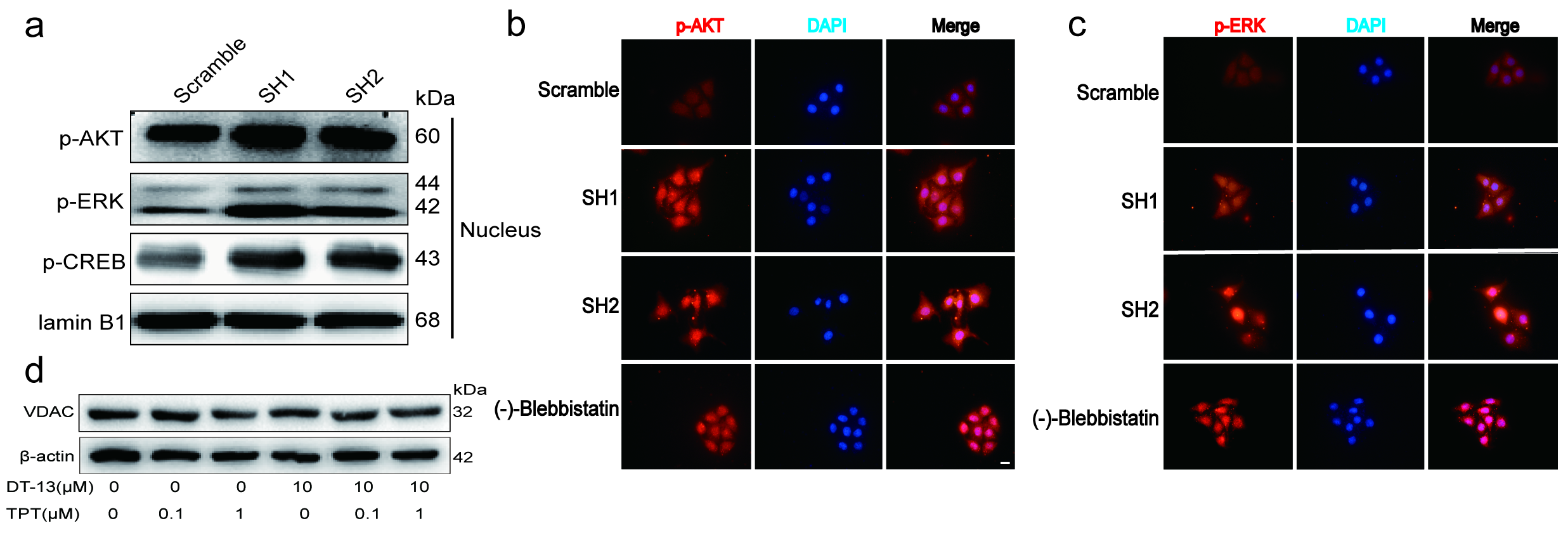


**Supplementary Figure S4: The effect of NM IIA on the activity of CREB in BGC-823 cells.** (a) The p-ERK, p-AKT and p-CREB levels in cell nucleus were detected using western blot analysis in NM IIA knock down BGC-823 cells. (b-c) The p-ERK and p-AKT levels in cell nucleus were detected using cell fluorescence analysis when NM IIA knock down or treated with (-)-blebbistatin in BGC-823 cells. (d) The VDAC level in whole cell lysate was detected by western blot analysis in BGC-823 cells.

**Supplementary Table S1**

Primer sequences for quantitative RT-PCR

| Gene | Species | Forward primer (5' to 3') | Reverse primer (5' to 3') |
| --- | --- | --- | --- |
| HK II | Human | GAGTTTGACCTGGATGTGGTTGC | CCTCCATGTAGCAGGCATTGCT |
| PKM2 | Human | ATGGCTGACACATTCCTGGAGC | CCTTCAACGTCTCCACTGATCG |
| LDHA | Human | GGATCTCCAACATGGCAGCCTT | AGACGGCTTTCTCCCTCTTGCT |
| PFK-1 | Human | GCTTCTAGCTCATGTCAGACCC | CCAATCCTCACAGTGGAGCGAA |
| GLUT1 | Human | TTGCAGGCTTCTCCAACTGGAC | CAGAACCAGGAGCACAGTGAAG |
| GLUT4 | Human | CCATCCTGATGACTGTGGCTCT | GCCACGATGAACCAAGGAATGG |
| PDH | Human | TTCTGGAGCCACTGCTTGTGTG | ACAGCGTGACTGCTGACCATGA |
